# The crosstalk between ferroptosis and cellular senescence in gestational diabetes mellitus: from iron dyshomeostasis to trophoblast dysfunction

**DOI:** 10.3389/fendo.2026.1975811

**Published:** 2026-10-30

**Authors:** Min Li, Wendi Zhao, Lei Jiang, Yijing Chu

**Affiliations:** 1 Department of Obstetrics, Qingdao University Affiliated Hospital, Qingdao, China; 2 Department of Gynecology, Qingdao University Affiliated Hospital, Qingdao, China

**Keywords:** cellular senescence, ferroptosis, fetal growth restriction, fetal overgrowth, gestational diabetes mellitus, iron homeostasis, trophoblast

## Abstract

Gestational diabetes mellitus (GDM) comprises heterogeneous placental phenotypes that include fetal overgrowth, large-for-gestational-age birth, and fetal growth restriction (FGR). We propose a testable endotype model in which maternal hyperglycemia, advanced glycation end products, diabetic dyslipidemia, altered polyunsaturated fatty-acid delivery, gestational iron exposure, and the physiologic fall in hepcidin converge on a redox-stressed trophoblast microenvironment. In proliferative cytotrophoblasts and extravillous trophoblasts, the relative reserve of GPX4 and FSP1/CoQ10, ACSL4-dependent PUFA-phospholipid loading, proliferative status, and insult intensity and duration are predicted to determine whether cells undergo ferroptosis, enter senescence, or occupy an intermediate state. We further propose an asymmetric crosstalk model: senescent trophoblasts are relatively ferroptosis-resistant, retain iron, and act in trans through SASP factors, reactive oxygen species, and oxidized lipids on ferroptosis-susceptible neighboring cells. The model predicts a gestational-age-dependent window in which physiologic senescence accumulates and its paracrine effects become injurious. Ferrostatin-1 or liproxstatin-1 should rescue lipid-peroxidation-driven death in susceptible cells without reversing senescence in iron-retaining cells. The model is GDM-specific in its initiating inputs and gestational context, but the reciprocal links remain hypotheses rather than established facts. It predicts that an injury-dominant placental endotype will show spatially adjacent iron-retaining senescence-like cells and lipid-peroxidation-driven cell death, impaired nutrient transport, and FGR, whereas compensatory transport and villous expansion may produce large-for-gestational-age phenotypes in other GDM placentas. We outline experiments that could support or falsify this model and define pregnancy-specific safety constraints for therapeutic testing.

## 1 Introduction

GDM is a common pregnancy complication in which maternal insulin resistance, hyperglycemia, dyslipidemia, and inflammation alter placental development and function (1–3). The clinical phenotype is not uniform: many pregnancies are associated with fetal overgrowth or large-for-gestational-age (LGA) birth, whereas a clinically important subgroup has placental insufficiency and FGR (4–7). Recent placental omics syntheses indicate marked molecular heterogeneity, with relatively few molecular alterations reproduced across cohorts (8–10). A mechanistic model should therefore be presented as an endotype hypothesis rather than a universal property of every GDM placenta.

The placenta is a particularly relevant site for this question because iron is required for mitochondrial respiration, DNA synthesis, and fetal erythropoiesis, while excess redox-active iron can amplify lipid peroxidation. Ferroptosis is an iron-dependent, phospholipid-peroxidation-driven form of regulated cell death (5–7). Cellular senescence is a durable stress-associated state with altered chromatin, lysosomal activity, and secretion of inflammatory mediators, but its interpretation in the placenta must be cell-type specific because normal trophoblast differentiation also involves terminal cell-cycle exit and substantial remodeling (9, 20, 22).

The central hypothesis of this article is that a GDM placental endotype can be created when disturbed iron handling, lipid-peroxidation pressure, and mitochondrial stress exceed the adaptive capacity of trophoblasts. The model is more specific than simply transplanting a general ferro-aging framework (11, 12) or the ferro-aging-like loop described in preeclampsia (13). It assigns distinct fates to distinct trophoblast compartments, makes the senescence-to-ferroptosis relationship asymmetric, and provides predictions that can be tested in matched human tissue, trophoblast cultures, placental explants, and pregnancy models.

## 2 Literature search strategy

For this non-systematic hypothesis synthesis, PubMed/Medline was searched from January 2012 through 23 September 2026 using combinations of gestational diabetes, maternal diabetes, placenta, trophoblast, iron, labile iron, ferroptosis, cellular senescence, lipid peroxidation, NCOA4, HO-1, NRF2, AGE-RAGE, and fetal growth restriction. Reference lists of key reviews and recent primary studies were screened for pregnancy-specific evidence. No formal risk-of-bias scoring or quantitative synthesis was attempted; the evidence map in **Table 1** is intended to make direct evidence and extrapolation visible.

**Table 1:** Key studies supporting, informing, or limiting the proposed model.

Study	Model and species	Evidence setting	Main finding	Principal limitation
Zaugg et al. (1)	Human placenta; GDM	Direct GDM	Transporter and regulator changes; total placental iron not increased; partly adaptive interpretation.	Labile iron pool and ferritinophagy were not directly measured.
Zhang et al. (2)	GDM/FGR placenta; HTR-8/SVneo; STZ mouse	Direct GDM	GLUT1-AMPK/ACC lipid metabolism and ferroptosis-associated FGR; Lip-1/WZB117 rescue.	Selected FGR subgroup; does not explain LGA or all GDM.
Kadam et al. (9)	Primary trophoblasts from type A2 GDM	Direct GDM	Differential p53/Lamin A/C and p16-Rb senescence programs; altered p21.	No direct ferroptosis-senescence causality.
Fang et al. (17)	GDM trophoblast models and pregnancy model	Direct GDM	Metformin-associated protection linked to Nrf2/HO-1 and ferroptosis-associated changes.	Pharmacologic association; pregnancy safety and pathway specificity remain open.
Monisha et al. (18)	Human GDM placenta	Direct GDM	Coordinated oxidative-stress, ferroptosis, inflammatory, and angiogenic marker changes.	Marker changes alone do not prove ferroptotic death.
Etchegaray-Armijo et al. (4)	Systematic review and meta-analysis	GDM	Oxidative-stress biomarkers are generally higher but heterogeneous across studies.	Heterogeneity and observational designs limit mechanistic inference.
Fakonti et al. (10)	Placental transcriptomic/proteomic studies	Maternal diabetes	Molecular heterogeneity and recurrent vascular, immune, and developmental alterations.	Cross-study heterogeneity; not a direct crosstalk test.
Maus et al. (11)	Non-placental disease models	Extrapolated	Iron accumulation can drive fibrosis, senescence, and SASP.	Does not establish the proposed GDM trophoblast loop.
Hu et al. (13)	Preeclampsia trophoblasts	Other pregnancy disorder	SIRT1-p53 ferro-aging-like program linked to trophoblast dysfunction.	Preeclampsia has a different initiating stress and clinical phenotype.
Zhang et al. (15, 16)	Preeclampsia trophoblasts	Other pregnancy disorder	DECR1, TfR1, lipid peroxidation, ferroptosis, and syncytialization provide pregnancy-specific mechanisms.	Not GDM; cell-type and disease context differ.
Wei et al. (14)	Senescent fibroblasts; diabetic wound	Non-placental	Defective NCOA4 ferritinophagy and relative ferroptosis resistance in senescent cells.	Supports asymmetric resistance, not bystander senescence in GDM.
Kataria et al. (24); van Santen et al. (25)	Human pregnancy cohorts/meta-analysis	Systemic pregnancy context	Maternal iron status associates with GDM risk; hepcidin falls in later pregnancy.	Ferritin is confounded by inflammation; placental labile iron remains unmeasured.

Direct GDM evidence is separated from evidence extrapolated from other pregnancy disorders or non-placental systems.

## 3 GDM-specific entry points into placental iron and redox stress

### 3.1 What is established and what remains unmeasured

Placental iron handling involves transferrin receptor 1 (TfR1), divalent metal transporter 1 (DMT1), ZIP8, ferroportin (FPN1), hephaestin, IRP1, ferritin, and systemic hepcidin (8, 23, 26, 27). In the human GDM study by Zaugg et al., transporter transcripts and proteins, cellular localization, and total placental iron were assessed. The authors interpreted the coordinated transporter changes as at least partly adaptive, potentially limiting iron-mediated damage; total placental iron was not increased in their cohort (1). Labile iron pool measurements were not reported. Current human GDM evidence therefore does not establish placental iron overload. The key unresolved variable is whether GDM changes the labile or spatially mismanaged iron pool, which should be measured directly using FerroOrange, calcein quenching, or related approaches.

This distinction matters because total tissue iron, ferritin abundance, transporter expression, and labile Fe^2+^ are not interchangeable measurements. A placenta can maintain total iron while changing subcellular partitioning, ferritin turnover, or the fraction of redox-active iron. The mechanistic claim is therefore conditional: if GDM trophoblasts show increased labile iron or altered ferritinophagy despite stable total iron, this would provide a plausible upstream link to lipid peroxidation; if labile iron is unchanged, the proposed axis would need to be revised toward lipid and mitochondrial mechanisms that are independent of iron accumulation.

### 3.2 Maternal iron status, hepcidin, and gestational context

Maternal iron status is both a potential modifier of placental injury and a possible upstream contributor to GDM. A meta-analysis associated higher ferritin, hemoglobin, transferrin saturation, and heme-iron exposure with GDM risk, although effect estimates were heterogeneous and observational confounding was substantial (24). Ferritin is an acute-phase reactant, and hepcidin is likewise induced by inflammatory signaling; neither should be interpreted as a stand-alone indicator of iron status in pregnancies complicated by obesity or chronic inflammation (8, 25). Interpretation should integrate soluble transferrin receptor, transferrin saturation, C-reactive protein, and related measures. Routine iron supplementation is standard obstetric care, and maternal hepcidin normally falls during the second and third trimesters to increase iron availability for maternal and fetal demands (25). The combination of supplementation, low gestational hepcidin, obesity-related inflammation, dietary heme iron, and a strong fetal iron sink could therefore alter the substrate available to a metabolically stressed placenta.

This context prevents a simple downstream-only model in which hyperglycemia causes iron dysregulation. The alternative possibility is a feed-forward metabolic susceptibility in which pre-existing maternal iron status or iron exposure modifies insulin resistance, placental iron trafficking, or the response to hyperglycemia. Studies should therefore record supplement dose and timing, dietary heme and nonheme iron, maternal ferritin, transferrin saturation, soluble transferrin receptor, hepcidin, C-reactive protein, BMI, and GDM treatment rather than treating placental iron as an isolated variable.

### 3.3 What is GDM-specific about the proposed model

The proposed GDM endotype differs from the ferro-aging framework described in other tissues and from the preeclampsia model in at least four ways. First, the initiating stress is chronic hyperglycemia and glycation, including AGE-RAGE signaling, rather than primarily intermittent hypoxia-reoxygenation. Second, maternal diabetic dyslipidemia and altered placental PUFA delivery may change the membrane substrate available for ACSL4- and ALOX15-dependent oxidation. Third, routine iron supplementation, low gestational hepcidin, and maternal-fetal iron partitioning create a distinctive iron exposure context. Fourth, the duration and timing of GDM differ across pregnancies and can interact with fetal growth trajectory, treatment, and placental maturation. These features are the proposed source of GDM-specificity; the ferroptosis-senescence crosstalk itself is not claimed to be novel in isolation.

## 4 Ferroptosis in GDM trophoblasts

Ferroptosis is best defined as a failure of the cellular systems that restrain iron-catalyzed phospholipid peroxidation. The relevant balance includes cystine uptake through system Xc-, glutathione synthesis, GPX4, FSP1/CoQ10, mitochondrial GPX4 and DHODH-associated defenses, PUFA incorporation into phospholipids through ACSL4 and LPCAT3, and oxidation by iron-dependent and enzymatic pathways including ALOX15 (5–8). This framework is broader than a list of GPX4, SLC7A11, ACSL4, ROS, MDA, or iron changes. Those measurements indicate a ferroptosis-associated state but do not by themselves prove ferroptotic death.

The strongest direct GDM evidence currently comes from the GLUT1 study in selected GDM placentas with FGR. That work combined human tissue, high-glucose trophoblast models, pharmacologic and genetic GLUT1 manipulation, and an STZ mouse model; inhibition of GLUT1 or treatment with liproxstatin-1 reduced ferroptosis-associated injury and improved the FGR phenotype (2). A separate GDM study linked metformin-associated protection to Nrf2/HO-1 signaling (17). Human GDM placental studies have also reported coordinated changes in oxidative-stress, ferroptosis, inflammatory, and angiogenic markers (18). These studies support a ferroptosis-relevant endotype, but only interventions that rescue the phenotype or genetic manipulation of the pathway can establish ferroptotic cell death.

The direction of the SIRT3 finding requires careful wording. One GDM placental report associated high glucose in trophoblast models with increased SIRT3, AMPK/mTOR signaling, and lower GPX4 (3). Because SIRT3 is often described as mitoprotective, this apparent directionality should be regarded as context-dependent and trophoblast-specific rather than a general property of SIRT3. Independent experiments should test whether SIRT3 is upstream of GPX4 loss, a compensatory response that becomes insufficient, or a marker of a different metabolic state.

## 5 Senescence and trophoblast subtype

Pathological senescence should be distinguished from normal differentiation and maturation. Cytotrophoblasts are mononuclear and proliferative, making durable arrest, EdU exclusion, p16/p21-Rb signaling, Lamin A/C remodeling, lipofuscin accumulation, and a persistent SASP biologically interpretable. Extravillous trophoblasts are also capable of proliferation and invasion and may show stress-induced arrest with altered inflammatory signaling. Syncytiotrophoblasts, by contrast, are terminally differentiated and multinucleated. A syncytium cannot be treated as a large mononuclear cell that has simply stopped cycling; SA-beta-gal, p16, or p21 positivity in this compartment requires interpretation alongside syncytialization markers, morphology, and secretory function.

The most direct GDM evidence for trophoblast senescence is the study of type A2 GDM pregnancies showing differential activation of p53/Lamin A/C and p16-Rb programs and disrupted p21-associated differentiation (9). Recent preeclampsia single-nucleus, spatial, and single-cell studies further emphasize senescent syncytiotrophoblast states, BHLHE40/NDRG1 programs, and non-cell-autonomous signaling, but these data cannot be relabeled as GDM evidence (20, 21). In the present model, the core senescence branch therefore refers primarily to cytotrophoblasts and extravillous trophoblasts. In syncytiotrophoblasts, the corresponding state is described more cautiously as stress-associated secretory, iron-retaining, or membrane-injury phenotypes unless a durable senescence program is independently established.

A minimum senescence panel should combine more than one canonical marker with functional evidence: SA-beta-gal or lipofuscin, p16INK4a and p21, Rb or Lamin A/C changes, long-term loss of proliferation, and a SASP panel including IL-6, IL-8, CCL2, TGF-beta, and selected matrix metalloproteinases. Ferritin, NCOA4, lysosomal function, and iron localization should be measured in parallel because senescent cells can alter iron storage without having a high labile iron pool.

## 6 A falsifiable asymmetric model of ferroptosis-senescence crosstalk

The central proposition is not that every stressed trophoblast enters both programs or that the two programs reinforce each other symmetrically. Instead, the model contains a shared upstream stress, a fate-selection threshold, and an asymmetric tissue-level feedback (5–8, 11–14). This proposed model, including the evidence coding and predicted trophoblast fate relationships, is summarized in **Figure 1**.

**Figure 1:**
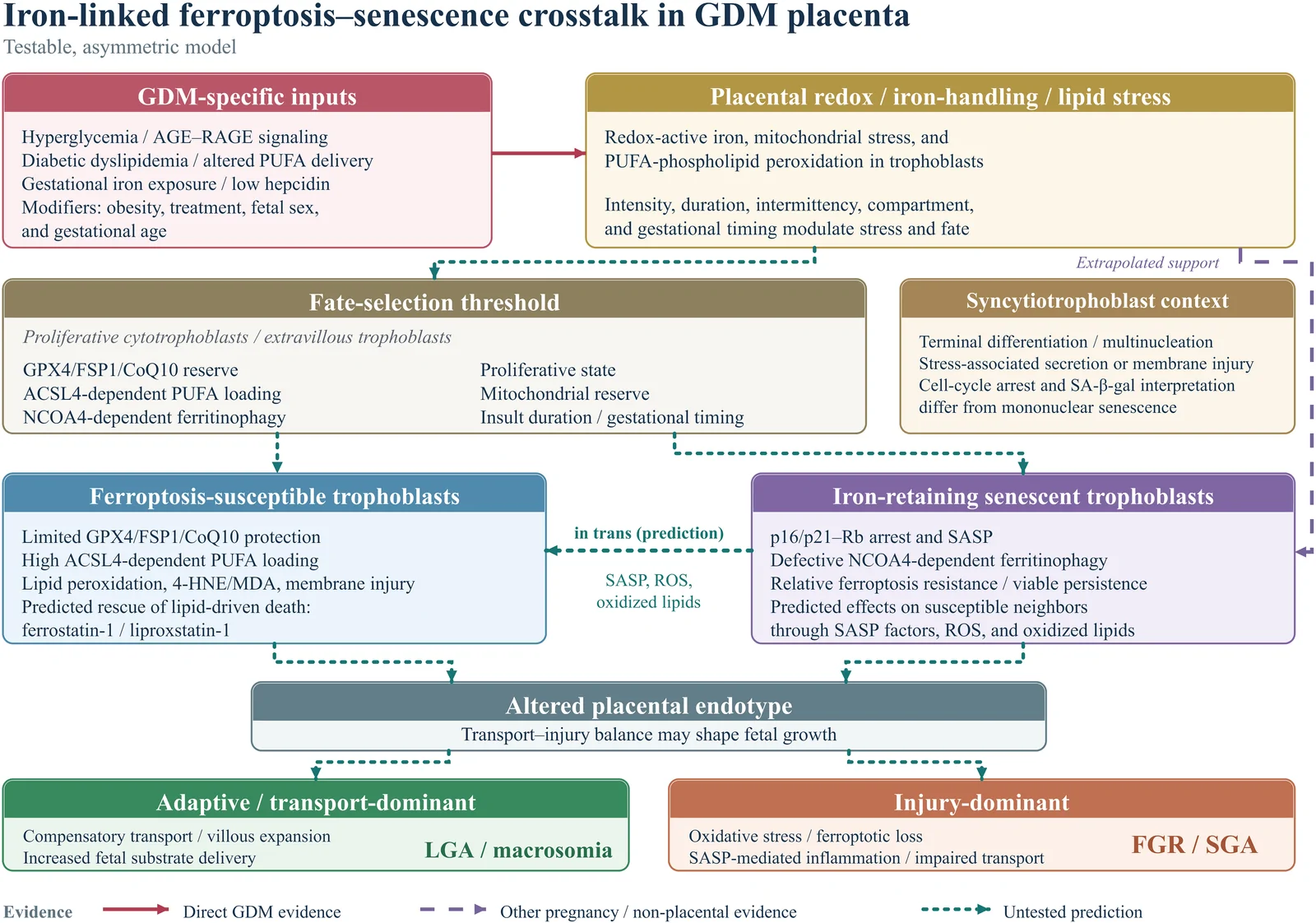
Testable, asymmetric model of iron-linked ferroptosis-senescence crosstalk in GDM placenta. GDM-specific inputs may converge on trophoblast redox, iron-handling, and lipid stress. In proliferative cytotrophoblasts and extravillous trophoblasts, the balance between lipid-peroxidation pressure and GPX4/FSP1 reserve, PUFA-phospholipid loading, proliferative state, insult duration, and gestational timing may determine ferroptosis, senescence, or coexistence. Senescent cells are predicted to retain iron and resist ferroptosis while acting in trans through SASP, ROS, and oxidized lipids on susceptible neighbors. Syncytiotrophoblasts are shown separately because terminal differentiation and multinucleation make cell-cycle arrest and SA-beta-gal interpretation non-equivalent to mononuclear trophoblast senescence. The dotted senescent-to-susceptible-cell arrow denotes an untested prediction, whereas the placental endotype may lead to LGA/macrosomia or FGR/SGA according to dose, timing, treatment, and compartment. Solid arrows indicate direct GDM evidence, long-dashed arrows indicate evidence from other pregnancy disorders or non-placental systems, and dotted arrows indicate untested predictions.

### 6.1 The fate threshold

Given a similar burden of iron, ROS, and lipid peroxidation, a trophoblast is predicted to undergo ferroptosis when peroxidation outruns GPX4/FSP1/CoQ10 and related defenses before a stable arrest program is established (5–8). It is predicted to enter senescence when DNA damage, mitochondrial stress, or inflammatory signaling crosses an arrest threshold while membrane-protective systems remain sufficient to prevent acute lipid-peroxidation-driven death (9, 11). Coexistence is possible when both thresholds are crossed, but the same cell should not be assumed to be simultaneously senescent and ferroptotic without time-resolved evidence.

The proposed determinants are residual GPX4 and FSP1 reserve; ACSL4, LPCAT3, and ALOX15 activity; PUFA-phospholipid composition; ferritin and NCOA4-dependent ferritinophagy; proliferative state; mitochondrial reserve; and the intensity, duration, and intermittency of the insult (2, 5–8, 14). This produces experimentally testable predictions. Increasing ACSL4 or PUFA-phospholipid loading should shift susceptible cytotrophoblasts toward ferroptosis, whereas preserving GPX4/FSP1 should shift them away from death. A prolonged lower-intensity insult should favor arrest and SASP, whereas an acute high-intensity insult should favor ferroptosis. The same perturbation can therefore yield different outcomes in a proliferative cytotrophoblast, an extravillous trophoblast, and a syncytiotrophoblast (2, 5–8, 14).

### 6.2 The asymmetric tissue-level feedback

Wei et al. reported that senescent fibroblasts have defective NCOA4-dependent ferritinophagy and are relatively resistant to ferroptosis, allowing iron-loaded senescent cells to persist in diabetic wounds (14). This finding supports a cell-autonomous resistance mechanism in senescent cells; it does not establish ferroptosis-induced bystander senescence.

Senescent cytotrophoblasts and extravillous trophoblasts are therefore proposed to be iron-retaining, ferroptosis-resistant sources of SASP factors, ROS, and oxidized lipids (11–14). Their main effect is predicted to occur in trans: they lower the ferroptotic threshold of neighboring non-senescent trophoblasts that still have a vulnerable PUFA-rich membrane pool. At present, the possibility that ferroptotic debris or oxidized lipids induce senescence in surviving neighbors remains an untested extrapolation. The p53 and iron-linked senescence literature supports the plausibility of iron-driven arrest (11–13), but it does not establish a reciprocal ferroptosis-to-senescence loop in GDM.

This asymmetric formulation generates a sharper spatial prediction than a reciprocal arrow: senescence markers and ferritin/NCOA4 changes should be enriched in persistent, viable cells, whereas C11-BODIPY oxidation, 4-HNE, MDA, GPX4 loss, and membrane injury should be enriched in adjacent ferroptosis-susceptible cells (2, 14, 20, 21). Ferrostatin-1 or liproxstatin-1 should rescue the latter population without necessarily reversing senescence in the former (2, 14). Conversely, a senomorphic or senolytic intervention should reduce neighboring lipid-peroxidation injury only if the effect is mediated through the senescent-cell secretome; it should not be expected to restore GPX4 in a cell that has already undergone ferroptotic death.

### 6.3 From placental endotype to fetal growth phenotype

The clinical endpoint must include both major GDM growth phenotypes. Moderate hyperglycemia or a compensatory placental response may increase nutrient transport, villous surface, or fetal substrate delivery and contribute to LGA/macrosomia. In an injury-dominant endotype, chronic oxidative stress, ferroptotic loss of vulnerable trophoblasts, senescence-associated inflammation, and villous architectural damage may reduce effective transport and contribute to FGR/SGA. The GLUT1 study provides direct mechanistic evidence for a ferroptosis-associated FGR subgroup (2), whereas recent experimental work links placental oxidative stress with fetal overgrowth in a high-fat-diet GDM model (19). These outcomes are not contradictory if the model includes dose, duration, gestational timing, fetal sex, maternal obesity, treatment, and trophoblast compartment as effect modifiers (2, 9, 19).

## 7 Evidence map

The evidence map (**Table 1**) indicates that the proposed model is supported by several complementary but unequally strong lines of evidence. Direct studies in GDM placentas and trophoblast models support disturbances in placental iron handling, trophoblast senescence, oxidative stress, and ferroptosis-associated injury, with the strongest mechanistic evidence currently linking the GLUT1-AMPK/ACC lipid-metabolism axis to ferroptosis-associated FGR. However, no study has yet directly demonstrated a reciprocal ferroptosis-senescence loop in GDM trophoblasts, and the labile iron pool remains largely unmeasured. Evidence from preeclampsia provides pregnancy- and trophoblast-specific support for lipid peroxidation, TfR1 regulation, ferroptosis, syncytialization, and senescence-related signaling, whereas studies in diabetic wounds and other non-placental systems primarily inform the proposed role of NCOA4-dependent ferritinophagy and ferroptosis resistance in senescent cells. The available literature therefore supports the biological plausibility of the model but also indicates that it may represent a heterogeneous placental endotype rather than a universal feature of GDM. Importantly, changes in ferroptosis- or senescence-associated markers should not be interpreted as proof of cell death or permanent senescence without functional rescue, genetic manipulation, spatial analysis, or longitudinal evidence.

## 8 Experimental program and therapeutic boundaries

### 8.1 Model systems and cohort design

The hypothesis should be tested in a tiered program. First, primary villous cytotrophoblasts should be isolated from matched GDM and control placentas with careful separation of cytotrophoblast and syncytiotrophoblast-associated fractions (1, 9). Second, placental explants should preserve local cell-cell signaling and allow spatial measurements (1, 9, 20). Third, HTR-8/SVneo and BeWo cells should be exposed to defined high-glucose conditions with osmolarity controls, physiological insulin, palmitate or linoleate where justified, and AGE-RAGE stimulation where relevant (2, 3, 17). Fourth, STZ and high-fat-diet pregnancy models should be used as complementary systems rather than interchangeable models (2, 19). Every human study should record gestational age, labor, mode of delivery, maternal BMI and obesity, GDM treatment (diet, insulin, metformin), A1 versus A2 classification, preeclampsia, fetal sex, birthweight percentile, and sample-processing interval (1, 4, 9, 10).

### 8.2 Readouts that separate the two fates

Ferroptosis should be assessed with C11-BODIPY oxidation, 4-HNE and MDA, GPX4, SLC7A11, FSP1, ACSL4, ALOX15, mitochondrial GPX4 or DHODH-related defenses, and labile iron measured by FerroOrange or calcein quenching (2, 5–8). Senescence should be assessed with SA-beta-gal, p16INK4a, p21, Rb, Lamin A/C, lipofuscin, EdU or long-term proliferation assays, and a SASP cytokine and chemokine panel (9, 11–14, 20, 21). Ferritin, NCOA4, lysosomal function, and iron localization should bridge the two panels (14). A marker-only study should be labeled as ferroptosis-associated or senescence-associated rather than as proof of either fate (2, 9, 20, 21).

### 8.3 Causal tests and falsification criteria

The causal tests should include ferrostatin-1 or liproxstatin-1, deferoxamine, GPX4 or ACSL4 manipulation, FSP1 or Nrf2/HO-1 modulation, NCOA4 manipulation, and senomorphic or senolytic interventions selected for trophoblast relevance (2, 14, 17, 26, 27). Under the hypothesis, ferroptosis-directed rescue should reduce lipid-peroxidation-driven death and improve transport in an injury-dominant endotype, while senescence-directed interventions should reduce SASP-mediated injury to neighboring cells. The model would be weakened or refuted if labile iron is unchanged across GDM and control samples, if ferroptosis inhibitors fail to rescue the phenotype despite adequate target engagement, if senescent cells are more ferroptosis-prone than neighboring cells, or if no spatial or conditioned-medium effect links senescent trophoblasts to injury in otherwise susceptible cells (22, 23, 26, 27).

Fixed human tissue requires special caution. Immunostaining for GPX4, ACSL4, ferritin, p16, p21, Lamin A/C, 4-HNE, and cell-type markers can establish a spatially resolved association, but no fixed-tissue marker panel alone can prove ferroptotic death or establish a persistent senescent state. The strongest tissue-level claim should therefore be phrased as a ferroptosis-associated injury or senescence-like program unless supported by orthogonal morphology, *ex vivo* functional assays, pharmacologic rescue, or genetic evidence from matched models (20, 21).

### 8.4 Therapeutic safety and timing

Therapeutic translation is constrained by pregnancy biology. Iron chelation can deprive the fetus of iron needed for erythropoiesis and neurodevelopment, and the fetus is a strong iron sink. Dasatinib, quercetin, and navitoclax have substantial fetal-safety concerns; ferrostatin-1 and liproxstatin-1 are experimental compounds rather than clinical-grade therapies (2, 17, 26, 27). Any preclinical intervention should define a gestational window, maternal and fetal iron status, placental cell-type selectivity, and effects on normal trophoblast differentiation and physiological senescence.

Lower-risk strategies may initially include optimization of glycemic and lipid exposure, limiting excess lipid peroxidation, or modulating NRF2/HO-1 and related antioxidant responses while preserving iron delivery. Metformin-associated reduction of ferroptosis-associated changes in trophoblast models is a useful proof of pharmacologic tractability, not evidence that direct ferroptosis inhibition is ready for clinical use (17). The therapeutic window should be judged by whether a treatment reduces pathological injury without suppressing the normal differentiation, endocrine, and transport functions of the placenta (17, 26, 27).

## 9 Conclusion

The revised hypothesis is that iron-linked ferroptosis-senescence crosstalk defines a subset of GDM placentas rather than a universal feature of GDM. Its GDM-specific inputs are chronic hyperglycemia and glycation, diabetic lipid delivery, maternal iron status and supplementation, suppressed gestational hepcidin, and a variable gestational time course. Its most testable mechanistic feature is asymmetry: viable senescent trophoblasts may retain iron and resist ferroptosis while their SASP, ROS, and oxidized lipids damage susceptible neighboring trophoblasts. The model predicts spatially adjacent but biologically distinct cell states, explains why the same clinical diagnosis can produce LGA/macrosomia or FGR/SGA, and provides explicit outcomes that would support or falsify the theory.

## Data Availability

The original contributions presented in the study are included in the article/supplementary material. Further inquiries can be directed to the corresponding author.
